# Supplementary material for: α-Copaene is a potent repellent against the Asian Citrus Psyllid Diaphorina citri
Source: Sci Rep. 2025 Jan 28;15:3564. doi: 10.1038/s41598-025-86369-1 (PMC11775201; doi:10.1038/s41598-025-86369-1)
Supplement: Supplementary file 1 — Supplementary Material 1 [file 41598_2025_86369_MOESM1_ESM.pdf]

Additional Information

Dataset with the raw data used to generate figures 2, 3, 4, and 5

Figure 2A		Figure 2B		Figure 2C	
**Caryophyllene**	**Control**	**Caryophyllene**	**Control**	**Caryophyllene**	**Control**
0	10	0.15	9.85	3.583333	6.416667
3.166667	6.833333	0.55	9.45	4.866667	5.133333
3.133333	6.866667	3.05	6.95	0	10
10	0	0	10	7.083333	2.916667
10	0	6.83	3.17	6.55	3.45
7.633333	2.366667	1.62	8.38	0.95	9.05
0	10	5.78	4.22	1.683333	8.316667
6.25	3.75	5.67	4.33	7.15	2.85
0	10	2.75	7.25	5.466667	4.533333
6.233333	3.766667	1.07	8.93	4.316667	5.683333
0	10	6.58	3.42	4.966667	5.033333
10	0	8.18	1.82	5.233333	4.766667
0	10	2.02	7.98	10	0
10	0	6.48	3.52	5.183333	4.816667
6.933333	3.066667	0	10	6.25	3.75
0.25	9.75	2.6	7.4	0.883333	9.116667
0	10	0.42	9.58	1.233333	8.766667
10	0	6.6	3.4	10	0
0	10	5.25	4.75	0.716667	9.283333
0	10	4.75	5.25	0.95	9.05
6.066667	3.933333	10	0	4.583333	5.416667
4.533333	5.466667	6.93	3.07	0.85	9.15
4.566667	5.433333	6.28	3.72	0.366667	9.633333
0	10	2.15	7.85	5.9	4.1
0	10	4.57	5.43	2.783333	7.216667
0	10	4.03	5.97	0	10
4.516667	5.483333	0	10	0	10
10	0	6.03	3.97	6.033333	3.966667
10	0	10	0	8.966667	1.033333
3.783333	6.216667	2.8	7.2	6.5	3.5
0	10	4.28	5.72	6.6	3.4

0	10	5.77	4.23	0.966667	9.033333
0.633333	9.366667	0	10	0	10
9.8	0.2	0	10	9.516667	0.483333
4.066667	5.933333	9.47	0.53	10	0
0	10	8.67	1.33	6.866667	3.133333
0	10	1.67	8.33	10	0
0.55	9.45	8.72	1.28	7.95	2.05
5.25	4.75	4.03	5.97	4.433333	5.566667
0	10	9.85	0.15	8.85	1.15
9.533333	0.466667	0.25	9.75	6.233333	3.766667
1.333333	8.666667	1.18	8.82	5.833333	4.166667
10	0	6.67	3.33	7.116667	2.883333
2.05	7.95	6.67	3.33	4.1	5.9
4.166667	5.833333	3.43	6.57	0.2	9.8
9.633333	0.366667	0	10	1.883333	8.116667
10	0	0	10	5.166667	4.833333
2.6	7.4	2.53	7.47	1.983333	8.016667
3.933333	6.066667	8.7	1.3	5.833333	4.166667
6.633333	3.366667	6.83	3.17	6.2	3.8
9.766667	0.233333	0.32	9.68	6.283333	3.716667
0	10	0.57	9.43	2.15	7.85
0	10	0.78	9.22	1.666667	8.333333
5.716667	4.283333	0.38	9.62	0	10
1.683333	8.316667	10	0	1.15	8.85
7.466667	2.533333	1.03	8.97	0.95	9.05
2.55	7.45	5.87	4.13	1.2	8.8
0	10	7.9	2.1	2.35	7.65
7.033333	2.966667	10	0	8.416667	1.583333
2.3	7.7	1.23	8.77	3	7
0	10	10	0	6.05	3.95
4.916667	5.083333	1.42	8.58	6.816667	3.183333
9.5	0.5	5.6	4.4	0	10
0	10	4.85	5.15	0	10

5.65	4.35	10	0	1.116667	8.883333
0	10	2.68	7.32	6.133333	3.866667
0.316667	9.683333	0.22	9.78	5.2	4.8
1.25	8.75	5.25	4.75	10	0
0.483333	9.516667	3.15	6.85	2.9	7.1
2.566667	7.433333	2.6	7.4	8.45	1.55
9.766667	0.233333	0.6	9.4	7.65	2.35
9.1	0.9	8.57	1.43	8.3	1.7
3.15	6.85	2.87	7.13	8.166667	1.833333
4.95	5.05	2.47	7.53	4.366667	5.633333
4.55	5.45	8.98	1.02	7.333333	2.666667
0	10	5	5	1.483333	8.516667
0	10	6.63	3.37	1.016667	8.983333
0.433333	9.566667	0	10	3.433333	6.566667
0	10	4.12	5.88	1.15	8.85
0.666667	9.333333	4.95	5.05	0.733333	9.266667
10	0	10	0	8.933333	1.066667
0.616667	9.383333	0	10	6.583333	3.416667
6.716667	3.283333	2.67	7.33	6.5	3.5
0	10	2.3	7.7	10	0
1.3	8.7	3.25	6.75	0	10
7.883333	2.116667	9.1	0.9	0.383333	9.616667
0.883333	9.116667	0	10	0	10
9.6	0.4	10	0	0	10
10	0	2.67	7.33	7.266667	2.733333
8.383333	1.616667	10	0	2.583333	7.416667
8.783333	1.216667	6.27	3.73	4.583333	5.416667
0	10	0	10	6.816667	3.183333
5.783333	4.216667	8.67	1.33	10	0
1.333333	8.666667	0	10	0	10
5.583333	4.416667	9.2	0.8	8.066667	1.933333
4.15	5.85	7.05	2.95	7.383333	2.616667
10	0	10	0	10	0

8.666667	1.333333	10	0	6.366667	3.633333
1.166667	8.833333	6.43	3.57	2.683333	7.316667
		10	0	0	10
		10	0	7.883333	2.116667
		10	0	10	0
		4.52	5.48	5.9	4.1
				8.1	1.9
				7.216667	2.783333
				0	10
				1.3	8.7
				2.85	7.15
				5.133333	4.866667
				5.783333	4.216667
				2.45	7.55

Figure 3A				
**Time (min)**	**Caryophyllene**			
	**Mean (xE8)**	**SEM**		
3	2.66	0.34		
6	2.31	0.37		
9	2.26	0.52		
12	2.36	0.46		
15	2.72	0.53		
**RAW DATA-Caryophyllene**				
3 min	6 min	9 min	12 min	15 min
8530447	11053310	15583612	8206448	9369241
5781608	7167303	4845425	5597182	6078842
10262541	2322740	4137058	8278420	10993271
9591658	4457877	8627768	11873616	11969859
6870316	6288357	9319968	6620649	7386032
7468203	6974767	6143931	4582375	5068477
4812725	9277120	4200200	4651644	6350825
10990160	6770081	4729570		
3399491	5078879	3305359		
	7418950			
	2932805			
**Figure 3B**				
**Time (min)**	**Humulene**			
**Mean (xE7)**	**SEM**			
3	1.42	0.21		
6	1.22	0.27		
9	1.05	0.25		
12	1.02	0.26		
15	1.19	0.34		
**RAW DATA-Humulene**				
3 min	6 min	9 min	12 min	15 min
305500185	403258211	574794931	224814575	256340259
197540726	445132247	123437089	183322059	212476080
407160640	94899358	146400646	217051713	308990266
301221722	131890232	230289298	502332751	565496076
256460085	218140452	377060736	234501654	265423365
233645645	187920970	207492286	132794916	148932586
143122708	344428638	115897680	159610252	149580804
414076762	214065801	177780460		
137938013	142385338	80677668		
	282708366			
	74775783			

**Figure 3C**				
**Time (min)**	**Copaene**			
**Mean (xE6)**	**SEM**			
3	7.52	0.85		
6	6.34	0.77		
9	6.77	1.3		
12	7.12	0.98		
15	8.17	0.99		
**RAW DATA-Copaene**				
3 min	6 min	9 min	12 min	15 min
15949721	19447413	27621691	8187679	9037903
9613980	33978572	5338108	9048133	8695074
24259965	4953987	7720424	7814254	12801520
14225953	5213910	8823094	25891561	31535093
13935996	10734379	17946672	8252990	9876338
10278038	7231610	7964407	5313738	6100143
8206612	17233237	5740921	7023177	5466575
23833860	9190591	10160059		
7150897	7169745	3398341		
	14546661			
	4221148			

Figure 4

Differential Residence Times							
**0.1 ng/ul**	**0.5 ng/ul**	**0.9 ng/ul**	**1.3 ng/ul**	**1.7 ng/ul**	**2.1 ng/ul**	**2.5 ng/ul**	**2.9 ng/ul**
7.22	2.06	-0.76	6.06	5.02	-4.4	-6.2	6.16
7.86	0.36	-3.42	-2.46	-3.1	-3.74	3.8	10
1.46	-3.72	-1.68	-2.54	-3.58	-6.88	10	3
2.18	-3.9	-2.8	-1.86	-10	1.22	2.58	-5.26
5.9	-2.76	5.4	5.5	-10	-10	-8.4	-5.82
-0.1	-1.42	10	6.5	1.5	0.02	-7.82	10
6.26	-0.22	-4.04	7.22	5.42	7.66	2.02	3.88
0.88	-5.1	10	9.26	3.76	-6.12	-1	-8.46
-1.66	2.9	-0.1	5.56	8.64	-10	7.96	-7.1
8.64	3.52	2.28	-9	-3.42	-10	5.66	-5.36
-0.84	-3.32	2.36	-10	10	5.8	-6.02	4.66
1.26	-2.04	-2.84	-7.4	10	0.16	0.36	9.88
-5.3	-10	0.96	-7.52	-4.06	6.8	-6.74	-5.94
-0.36	7.54	7.58	6.4	-1.66	6.14	8.14	2.82
-1.38	-7.28	4.78	10	-3.14	0.42	-0.86	-7.12
-2.72	-9.04	1.26	-8.5	10	4.32	-1.68	-1.08
6.28	10	-3.94	0.9	4.4	-0.04	1.34	-1.1
1.04	-1.8	8.38	1.58	7	-6.56	-1.86	1.94
-4.7	7.9	3.22	3.76	-9.94	3.92	-3.26	6.28
-1.88	-1.06	4.04	-3.28	-2.38	-0.48	6.2	2.26
2.98	3.62	2.42	4.4	4.52	6.06	-5.12	4.82
0.4	5.12	4.32	7.9	5.34	9.9	6.6	-9.52
2.6	4.28	-6.3	10	2.14	-3.7	3.92	4.2
-2.64	5.933334	10	10	10	-1.68	10	4.82
-1.14	7.24	-0.08	-0.06	0.98	4.7	3.36	-3.84
10	-3.8	3.22	-4.24	2.86	6.96	-4.58	-2.46
-8.18	7.82	-4.38	-4.62	-4.84	5.78	10	1.84
-5.84	-4.54	0.72	6.02	0.14	2.14	10	8.28
10	1.26	3.06	4.6	5.94	8.42	-10	5.88
-10	-5.32	5.88	-5.88	5.8	10	-6.32	2.84

-4.86	-4.06	-10	-10	3.04	-6.64	-9.3	7.3
-3.08	8.4	10	-8.1	1.82	-8.44	5.56	-10
4.16	-5.52	10	-0.26	-6.56	-2.38	10	10
-6.3	-0.08	4.3	5.66	2.08	-2.38	7.9	-5.84
10	10	10	5.52	-6.32	-1.06	0.84	-10
-1.9	-0.54	-2.64	9.64	7.14	-10	8.24	-6.48
-1.54	-1.82	7.6	-10	2.14	6.54	-8.1	-4.52
0.54	0.38	6.54	9.78	10	5.62	5.98	-5.98
10	0.9	4.76	8.2	-10	4.04	1.18	-7.48
-0.44	-2.72	-6.74	6	-5.56	7.02	-7.92	0.88
9.2	-2.72	2.86	-10	-7.73333	4.08	9.4	10
1.86	-5.36	8.34	1.78	0.66	8.56	8.26	-0.42
-4.06	-3.26	-5.46	3.86	10	-0.32	5.46	2.78
-0.26	-0.1	-2.68	2.14	-8.56	8.2	-6.68	4.12
-7.62	4.3	2.28	7.18	10	-4.76	-5.84	2.18
1.64	-10	-5.14	2.28	-4.84	7.88	-4.84	0.54
9.84	4.4	3.88	0.5	-10	-8.26	-1.94	1.78
-2.88	-3.32	0.52	5.1	7.78	-10	-10	-2.92
-10	10	8.6	-5.2	-6.64	6.82	-3.88	-4.84
5.82	10	10	-3.88	2.56	-5.94	4.12	-4.64
-7.52	-2.18	10	6.28	2.22	8.42	-3.84	2.88
-2.88	5.94	-2.08	10	-0.68	4.56	-4.7	-1.28
-4.36	3.44	4.52	5	10	3.46	7.8	1.04
7.56	0.06	9.5	-10	3.82	10	3.7	9.7
-6.26	10	8.16	-10	4.68	-3.7	-7.56	1.58
-9.24	10	-8.42	-5.44	10	-5.06	1.24	-9.84
-8.86	-5.58	10	-5.44	1.62	-4.04	-1.96	-3.2
0.88	10	-10	10	4.82	6.38	3.34	4.92
4.4	-10	-4.62	10	6.72	4	-10	0.16
7.08	3.86	6.88	-10	-1	8.5	1.34	5
1.14	5.84	1.21	4.48	7.1	-8.82	-10	-2.24
-6.44	-5.26	10	0.08	10	-3.54	-2.66	-2.52
0.9	-1.98	-5.46	0.94	10	-2.08	-2.02	0.94

-4.92	-4.08	-3.82	-5.42	-10	-0.76	-3.26	-2.9
-1.8	-3.02	-6.36	5.72	7.02	7.66	10	0.48
0.58	-0.16	6.24	5.16	-9.82	5.92	-2.24	6.4
-1.48	-2	3.82	1.42	2.06	4.1	7.16	-4.76
1.78	5.8	-4.4	5.9	-5.04	0.26	3.18	4.5
-4.08	-10	-5.02	9.6	-10	5.16	1.68	-2.18
4.4	10	4.12	-9.34	-8.78	-1.3	1.34	-10
-4.02	5.24	-4.02	-2.3	1.82	-3.72	-10	-3
0.38	-10	2.48	7.52	10	-5.38	-8.02	-0.88
0.26	-0.52	6.82	8.34	-3.4	2.28	-0.58	-7.04
6.1	-6	2.22	4.62	1.08	-10	-10	-5.8
10	5.86	-5.96	2.52	10	6.46	-1.64	-10
-3.42	-1.06	8.4	-5.02	10	-10	-6.12	10
1.76	7.86	1.5	2.44	10	0.06	-3.42	-0.06
3.58	-7.68	-4.6	6.54	-4.98	5.38	0.5	-6.24
-3.06	-1.86	2.84	3.66	-7.06	10	-1.36	-10
1.96	-6.18	-2.06	7.7	7.4	10	5.96	-1.04
10	-3.78	-10	10	-7.41	-4.46	3.64	-6.8
-3.3	-4.78	-4.82	7.1	6.26	0.62	-0.9	-10
8.48	-4.08	-1.46	-1.98	-3.48	0.94	6.76	-10
-4.42	3.88	7.24	4.32	10	-4.22	-1.92	4.14
1.56	10	-4.66	2.9	8.62	-3	-4.42	-6.42
-8.2	-6.42	-0.32	3.5	10	7.3	-4.3	6.46
10	-2.76	8.1	0.24	10	10	7.58	4.24
8.16	-3.46	-6.28	-2.42	3.82	3.86	9.96	3.32
10	-8.36	-3.52	-7.72	-5.84	6.76	8.76	-1.72
-0.28	-5.24	-4.68	8.76	1.924	-0.1	-10	-8.96
5.12	10	2.8	6.7	3.52	-8.22	2.76	0.64
5.22	10	8.08	3.9	-5.62	5.38	-2.44	6.58
-3.62	4.24	1.94	10	-1.22	7.96	7.62	-1.88
0.56	-8.14	-2.16	0.24	-5.54	8.1	-1.96	9.28
-1.9	-2.72	8.9	8.46	9.5	6.76	-3.5	10
-0.54	9.12	4.62	-2.98	6.66	6.08	2.58	-7.62

-6.46	2.06	10	-7.02	-0.16	-10	6.3	5.16
0.32	1.4	-4.26	10	3.38	-5.8	-0.2	-10
3.16	-2.14	-3.22	9.18	0.32	6.3	2.14	7.48
	4.1	5.22	4.68	3.2	10	10	4.62
	-1.92	4.58	10	10	7.62	10	-10
	-3.92	-6.04	-2.16	2.02	3.04	5.16	8.26
	7.66	8.24	5.78	-8.2	10	0.46	2.02
	-1.8	-6.04	10	2.94	10	10	
	-5.82	0.16	10	-2.06	6	-0.06	
	0.84	6.48	-8.12	-0.98	6.04	10	
	7.78	-4.52	2.44		3.22	6.32	
		-0.06	-2.94		3.12		
		-0.08	-10		-5.88		
		-5.98	-9.84		0.62		
		7.92	-0.82		8.32		
		2.28	-1.72		6.74		
		5.98	-3.76		-10		
		-3.12	-0.14		-7.7		
		4.1	-3.1		-3.46		
		-2.16	9.14		3.24		
		1.98	-1.32		0.66		
		-3.58	-1.86				
		2.06	-0.06				
		-6.48	-5.94				
		0.42	-3.34				
		4.9	2.36				
		10	1.72				
		6.02	7.5				
		-4.6	10				
		9.92	6.04				
		-9.58					
		9.02					
		6.92					

RAW DATA

0.1 ng/ul	CONTROL	0.5 ng/ul	CONTROL	0.9 ng/ul	CONTROL	1.3 ng/ul	CONTROL
1.39	8.61	3.97	6.03	5.38	4.62	1.97	8.03
1.07	8.93	4.82	5.18	6.71	3.29	6.23	3.77
4.27	5.73	6.86	3.14	5.84	4.16	6.27	3.73
3.91	6.09	6.95	3.05	6.4	3.6	5.93	4.07
2.05	7.95	6.38	3.62	2.3	7.7	2.25	7.75
5.05	4.95	5.71	4.29	0	10	1.75	8.25
1.87	8.13	5.11	4.89	7.02	2.98	1.39	8.61
4.56	5.44	7.55	2.45	0	10	0.37	9.63
5.83	4.17	3.55	6.45	5.05	4.95	2.22	7.78
0.68	9.32	3.24	6.76	3.86	6.14	9.5	0.5
5.42	4.58	6.66	3.34	3.82	6.18	10	0
4.37	5.63	6.02	3.98	6.42	3.58	8.7	1.3
7.65	2.35	10	0	4.52	5.48	8.76	1.24
5.18	4.82	1.23	8.77	1.21	8.79	1.8	8.2
5.69	4.31	8.64	1.36	2.61	7.39	0	10
6.36	3.64	9.52	0.48	4.37	5.63	9.25	0.75
1.86	8.14	0	10	6.97	3.03	4.55	5.45
4.48	5.52	5.9	4.1	0.81	9.19	4.21	5.79
7.35	2.65	1.05	8.95	3.39	6.61	3.12	6.88
5.94	4.06	5.53	4.47	2.98	7.02	6.64	3.36
3.51	6.49	3.19	6.81	3.79	6.21	2.8	7.2
4.8	5.2	2.44	7.56	2.84	7.16	1.05	8.95
3.7	6.3	2.86	7.14	8.15	1.85	0	10
6.32	3.68	2.033333	7.966667	0	10	0	10
5.57	4.43	1.38	8.62	5.04	4.96	5.03	4.97
0	10	6.9	3.1	3.39	6.61	7.12	2.88
9.09	0.91	1.09	8.91	7.19	2.81	7.31	2.69
7.92	2.08	7.27	2.73	4.64	5.36	1.99	8.01

0	10	4.37	5.63	3.47	6.53	2.7	7.3
10	0	7.66	2.34	2.06	7.94	7.94	2.06
7.43	2.57	7.03	2.97	10	0	10	0
6.54	3.46	0.8	9.2	0	10	9.05	0.95
2.92	7.08	7.76	2.24	0	10	5.13	4.87
8.15	1.85	5.04	4.96	2.85	7.15	2.17	7.83
0	10	0	10	0	10	2.24	7.76
5.95	4.05	5.27	4.73	6.32	3.68	0.18	9.82
5.77	4.23	5.91	4.09	1.2	8.8	10	0
4.73	5.27	4.81	5.19	1.73	8.27	0.11	9.89
0	10	4.55	5.45	2.62	7.38	0.9	9.1
5.22	4.78	6.36	3.64	8.37	1.63	2	8
0.4	9.6	6.36	3.64	3.57	6.43	10	0
4.07	5.93	7.68	2.32	0.83	9.17	4.11	5.89
7.03	2.97	6.63	3.37	7.73	2.27	3.07	6.93
5.13	4.87	5.05	4.95	6.34	3.66	3.93	6.07
8.81	1.19	2.85	7.15	3.86	6.14	1.41	8.59
4.18	5.82	10	0	7.57	2.43	3.86	6.14
0.08	9.92	2.8	7.2	3.06	6.94	4.75	5.25
6.44	3.56	6.66	3.34	4.74	5.26	2.45	7.55
10	0	0	10	0.7	9.3	7.6	2.4
2.09	7.91	0	10	0	10	6.94	3.06
8.76	1.24	6.09	3.91	0	10	1.86	8.14
6.44	3.56	2.03	7.97	6.04	3.96	0	10
7.18	2.82	3.28	6.72	2.74	7.26	2.5	7.5
1.22	8.78	4.97	5.03	0.25	9.75	10	0
8.13	1.87	0	10	0.92	9.08	10	0
9.62	0.38	0	10	9.21	0.79	7.72	2.28
9.43	0.57	7.79	2.21	0	10	7.72	2.28
4.56	5.44	0	10	10	0	0	10
2.8	7.2	10	0	7.31	2.69	0	10
1.46	8.54	3.07	6.93	1.56	8.44	10	0
4.43	5.57	2.08	7.92	4.38	5.59	2.76	7.24

8.22	1.78	7.63	2.37	0	10	4.96	5.04
4.55	5.45	5.99	4.01	7.73	2.27	4.53	5.47
7.46	2.54	7.04	2.96	6.91	3.09	7.71	2.29
5.9	4.1	6.51	3.49	8.18	1.82	2.14	7.86
4.71	5.29	5.08	4.92	1.88	8.12	2.42	7.58
5.74	4.26	6	4	3.09	6.91	4.29	5.71
4.11	5.89	2.1	7.9	7.2	2.8	2.05	7.95
7.04	2.96	10	0	7.51	2.49	0.2	9.8
2.8	7.2	0	10	2.94	7.06	9.67	0.33
7.01	2.99	2.38	7.62	7.01	2.99	6.15	3.85
4.81	5.19	10	0	3.76	6.24	1.24	8.76
4.87	5.13	5.26	4.74	1.59	8.41	0.83	9.17
1.95	8.05	8	2	3.89	6.11	2.69	7.31
0	10	2.07	7.93	7.98	2.02	3.74	6.26
6.71	3.29	5.53	4.47	0.8	9.2	7.51	2.49
4.12	5.88	1.07	8.93	4.25	5.75	3.78	6.22
3.21	6.79	8.84	1.16	7.3	2.7	1.73	8.27
6.53	3.47	5.93	4.07	3.58	6.42	3.17	6.83
4.02	5.98	8.09	1.91	6.03	3.97	1.15	8.85
0	10	6.89	3.11	10	0	0	10
6.65	3.35	7.39	2.61	7.41	2.59	1.45	8.55
0.76	9.24	7.04	2.96	5.73	4.27	5.99	4.01
7.21	2.79	3.06	6.94	1.38	8.62	2.84	7.16
4.22	5.78	0	10	7.33	2.67	3.55	6.45
9.1	0.9	8.21	1.79	5.16	4.84	3.25	6.75
0	10	6.38	3.62	0.95	9.05	4.88	5.12
0.92	9.08	6.73	3.27	8.14	1.86	6.21	3.79
0	10	9.18	0.82	6.76	3.24	8.86	1.14
5.14	4.86	7.62	2.38	7.34	2.66	0.62	9.38
2.44	7.56	0	10	3.6	6.4	1.65	8.35
2.39	7.61	0	10	0.96	9.04	3.05	6.95
6.81	3.19	2.88	7.12	4.03	5.97	0	10
4.72	5.28	9.07	0.93	6.08	3.92	4.88	5.12

5.95	4.05	6.36	3.64	0.55	9.45	0.77	9.23
5.27	4.73	0.44	9.56	2.69	7.31	6.49	3.51
8.23	1.77	3.97	6.03	0	10	8.51	1.49
4.84	5.16	4.3	5.7	7.13	2.87	0	10
3.42	6.58	6.07	3.93	6.61	3.39	0.41	9.59
		2.95	7.05	2.39	7.61	2.66	7.34
		5.96	4.04	2.71	7.29	0	10
		6.96	3.04	8.02	1.98	6.08	3.92
		1.17	8.83	0.88	9.12	2.11	7.89
		5.9	4.1	8.02	1.98	0	10
		7.91	2.09	4.92	5.08	0	10
		4.58	5.42	1.76	8.24	9.06	0.94
		1.11	8.89	7.26	2.74	3.78	6.22
				5.03	4.97	6.47	3.53
				5.04	4.96	10	0
				7.99	2.01	9.92	0.08
				1.04	8.96	5.41	4.59
				3.86	6.14	5.86	4.14
				2.01	7.99	6.88	3.12
				6.56	3.44	5.07	4.93
				2.95	7.05	6.55	3.45
				6.08	3.92	0.43	9.57
				4.01	5.99	5.66	4.34
				6.79	3.21	5.93	4.07
				3.97	6.03	5.03	4.97
				8.24	1.76	7.97	2.03
				4.79	5.21	6.67	3.33
				2.55	7.45	3.82	6.18
				0	10	4.14	5.86
				1.99	8.01	1.25	8.75
				7.3	2.7	0	10
				0.04	9.96	1.98	8.02
				9.79	0.21		

				0.49	9.51		
				1.54	8.46		

1.7 ng/ul	CONTROL	2.1 ng/ul	CONTROL	2.5 ng/ul	CONTROL	2.9 ng/ul	CONTROL
2.49	7.51	7.2	2.8	8.1	1.9	1.92	8.08
6.55	3.45	6.87	3.13	3.1	6.9	0	10
6.79	3.21	8.44	1.56	0	10	3.5	6.5
10	0	4.39	5.61	3.71	6.29	7.63	2.37
10	0	10	0	9.2	0.8	7.91	2.09
4.25	5.75	4.99	5.01	8.91	1.09	0	10
2.29	7.71	1.17	8.83	3.99	6.01	3.06	6.94
3.12	6.88	8.06	1.94	5.5	4.5	9.23	0.77
0.68	9.32	10	0	1.02	8.98	8.55	1.45
6.71	3.29	10	0	2.17	7.83	7.68	2.32
0	10	2.1	7.9	8.01	1.99	2.67	7.33
0	10	4.92	5.08	4.82	5.18	0.06	9.94
7.03	2.97	1.6	8.4	8.37	1.63	7.97	2.03
5.83	4.17	1.93	8.07	0.93	9.07	3.59	6.41
6.57	3.43	4.79	5.21	5.43	4.57	8.56	1.44
0	10	2.84	7.16	5.84	4.16	5.54	4.46
2.8	7.2	5.02	4.98	4.33	5.67	5.55	4.45
1.5	8.5	8.28	1.72	5.93	4.07	4.03	5.97
9.97	0.03	3.04	6.96	6.63	3.37	1.86	8.14
6.19	3.81	5.24	4.76	1.9	8.1	3.87	6.13
2.74	7.26	1.97	8.03	7.56	2.44	2.59	7.41
2.33	7.67	0.05	9.95	1.7	8.3	9.76	0.24
3.93	6.07	6.85	3.15	3.04	6.96	2.9	7.1
0	10	5.84	4.16	0	10	2.59	7.41
4.51	5.49	2.65	7.35	3.32	6.68	6.92	3.08
3.57	6.43	1.52	8.48	7.29	2.71	6.23	3.77
7.42	2.58	2.11	7.89	0	10	4.08	5.92
4.93	5.07	3.93	6.07	0	10	0.86	9.14
2.03	7.97	0.79	9.21	10	0	2.06	7.94

2.1	7.9	0	10	8.16	1.84	3.58	6.42
3.48	6.52	8.32	1.68	9.65	0.35	1.35	8.65
4.09	5.91	9.22	0.78	2.22	7.78	10	0
8.28	1.72	6.19	3.81	0	10	0	10
3.96	6.04	6.19	3.81	1.05	8.95	7.92	2.08
8.16	1.84	5.53	4.47	4.58	5.42	10	0
1.93	9.07	10	0	0.88	9.12	8.24	1.76
3.93	6.07	1.73	8.27	9.05	0.95	7.26	2.74
0	10	2.19	7.81	2.01	7.99	7.99	2.01
10	0	2.98	7.02	4.41	5.59	8.74	1.26
7.78	2.22	1.49	8.51	8.96	1.04	4.56	5.44
8.866667	1.133333	2.96	7.04	0.3	9.7	0	10
4.67	5.33	0.72	9.28	0.87	9.13	5.21	4.79
0	10	5.16	4.84	2.27	7.73	3.61	6.39
9.28	0.72	0.9	9.1	8.34	1.66	2.94	7.06
0	10	7.38	2.62	7.92	2.08	3.91	6.09
7.42	2.58	1.06	8.94	7.42	2.58	4.73	5.27
10	0	9.13	0.87	5.97	4.03	4.11	5.89
1.11	8.89	10	0	10	0	6.46	3.54
8.32	1.68	1.59	8.41	6.94	3.06	7.42	2.58
3.72	6.28	7.97	2.03	2.94	7.06	7.32	2.68
3.89	6.11	0.79	9.21	6.92	3.08	3.56	6.44
5.34	4.66	2.72	7.28	7.35	2.65	5.64	4.36
0	10	3.27	6.73	1.1	8.9	4.48	5.52
3.09	6.91	0	10	3.15	6.85	0.15	9.85
2.66	7.34	6.85	3.15	8.78	1.22	4.21	5.79
0	10	7.53	2.47	4.38	5.62	9.92	0.08
4.19	5.81	7.02	2.98	5.98	4.02	6.6	3.4
2.59	7.41	1.81	8.19	3.33	6.67	2.54	7.46
1.64	8.36	3	7	10	0	4.92	5.08
5.5	4.5	0.75	9.25	4.33	5.67	2.5	7.5
1.45	8.55	9.41	0.59	10	0	6.12	3.88
0	10	6.77	3.23	6.33	3.67	6.26	3.74

0	10	6.04	3.96	6.01	3.99	4.53	5.47
10	0	5.38	4.62	6.63	3.37	6.45	3.55
1.49	8.51	1.17	8.83	0	10	4.76	5.24
9.91	0.09	2.04	7.96	6.12	3.88	1.8	8.2
3.97	6.03	2.95	7.05	1.42	8.58	7.38	2.62
7.52	2.48	4.87	5.13	3.41	6.59	2.75	7.25
10	0	2.42	7.58	4.16	5.84	6.09	3.91
9.39	0.61	5.65	4.35	4.33	5.67	10	0
4.09	5.91	6.86	3.14	10	0	6.5	3.5
0	10	7.69	2.31	9.01	0.99	5.44	4.56
6.7	3.3	3.86	6.14	5.29	4.71	8.52	1.48
4.46	5.54	10	0	10	0	7.9	2.1
0	10	1.77	8.23	5.82	4.18	10	0
0	10	10	0	8.06	1.94	0	10
0	10	4.97	5.03	6.71	3.29	5.03	4.97
7.49	2.51	2.31	7.69	4.75	5.25	8.12	1.88
8.53	1.47	0	10	5.68	4.32	10	0
1.3	8.7	0	10	2.02	7.98	5.52	4.48
8.44	1.03	7.23	2.77	3.18	6.82	8.4	1.6
1.87	8.13	4.69	5.31	5.45	4.55	10	0
6.74	3.26	4.53	5.47	1.62	8.38	10	0
0	10	7.11	2.89	5.96	4.04	2.93	7.07
0.69	9.31	6.5	3.5	7.21	2.79	8.21	1.79
0	10	1.35	8.65	7.15	2.85	1.77	8.23
0	10	0	10	1.21	8.79	2.88	7.12
3.09	6.91	3.07	6.93	0.02	9.98	3.34	6.66
7.92	2.08	1.62	8.38	0.62	9.38	5.86	4.14
4.038	5.962	5.05	4.95	10	0	9.48	0.52
3.24	6.76	9.11	0.89	3.62	6.38	4.68	5.32
7.81	2.19	2.31	7.69	6.22	3.78	1.71	8.29
5.61	4.39	1.02	8.98	1.19	8.81	5.94	4.06
7.77	2.23	0.95	9.05	5.98	4.02	0.36	9.64
0.25	9.75	1.62	8.38	6.75	3.25	0	10

1.67	8.33	1.96	8.04	3.71	6.29	8.81	1.19
5.08	4.92	10	0	1.85	8.15	2.42	7.58
3.31	6.69	7.9	2.1	5.1	4.9	10	0
4.84	5.16	1.85	8.15	3.93	6.07	1.26	8.74
3.4	6.6	0	10	0	10	2.69	7.31
0	10	1.19	8.81	0	10	10	0
3.99	6.01	3.48	6.52	2.42	7.58	0.87	9.13
9.1	0.9	0	10	4.77	5.23	3.99	6.01
3.53	6.47	0	10	0	10		
6.03	3.97	2	8	5.03	4.97		
5.49	4.51	1.98	8.02	0	10		
		3.39	6.61	1.84	8.16		
		3.44	6.56				
		7.94	2.06				
		4.69	5.31				
		0.84	9.16				
		1.63	8.37				
		10	0				
		8.85	1.15				
		6.73	3.27				
		3.38	6.62				
		4.67	5.33				

Figure 5

Caryophyllene 0.17 ug/ul +Copaene 1.7 ng/ul

Treatment	Control
2.53	7.47
8.63	1.37
2.45	7.55
10	0
7.8	2.2
5.95	4.05
2.84	7.16
6.56	3.44
10	0
1.07	8.93
4.83	5.17
7.3	2.7
8.1	1.9
4.03	5.97
0	10
0	10
3.95	6.05
3.71	6.29
0	10
2.82	7.18
4.14	5.86
1.95	8.05
4	6
10	0
4.02	5.98
9.95	0.05
1.22	8.78
9.68	0.32
10	0
2.85	7.15
0	10
0	10
5.22	4.78
2.84	7.16
4.11	5.89
10	0
10	0
7.55	2.45
1.5	8.5
8.77	1.23
5.07	4.93

Caryophyllene 0.13 ug/ul +Copaene 1.3 ng/ul

Treatment	Control
9.75	0.25
9.39	0.61
9.23	0.77
9	1
8.98	1.02
8.78	1.22
8.49	1.51
8.45	1.55
8.28	1.72
8.02	1.98
7.91	2.09
7.89	2.11
7.84	2.16
7.73	2.27
7.7	2.3
7.5	2.5
7.46	2.54
7.31	2.69
7.2	2.8
7.02	2.98
7.01	2.99
6.71	3.29
6.7	3.3
6.61	3.39
6.6	3.4
6.34	3.66
6.29	3.71
6.2	3.8
6.18	3.82
6.1	3.9
6.05	3.95
6.02	3.98
6	4
5.84	4.16
5.73	4.27
5.68	4.32
5.57	4.43
5.54	4.46
5.53	4.47
5.44	4.56
5.22	4.78

4.86	5.14
2.12	7.88
2.96	7.04
9.62	0.38
0	10
2.7	7.3
4.6	5.4
4.98	5.02
9.13	0.87
4.55	5.45
4.37	5.63
3.88	6.12
0.38	9.62
0	10
5.08	4.92
8.07	1.93
3.11	6.89
6.03	3.97
5.24	4.76
4.11	5.89
5.38	4.62
7.59	2.41
10	0
3.58	6.42
3.57	6.43
4.1	5.9
6.9	3.1
5.77	4.23
4.51	5.49
8.86	1.14
8.59	1.41
8.34	1.66
4.23	5.77
6.47	3.53
6.19	3.81
4.59	5.41
6.08	3.92
9.36	0.64
1.96	8.04
10	0
6.99	3.01
2.82	7.18
3.8	6.2
7.48	2.52
6.68	3.32

5.12	4.88
5.05	4.95
4.92	5.08
4.83	5.17
4.77	5.23
4.73	5.27
4.73	5.27
4.63	5.37
4.58	5.42
4.52	5.48
4.46	5.54
4.34	5.66
4.29	5.71
4.21	5.79
4.13	5.87
4.04	5.96
4.01	5.99
3.96	6.04
3.89	6.11
3.86	6.14
3.81	6.19
3.8	6.2
3.73	6.27
3.71	6.29
3.68	6.32
3.65	6.35
3.63	6.37
3.61	6.39
3.6	6.4
3.56	6.44
3.52	6.48
3.29	6.71
3.14	6.86
3.05	6.95
3.02	6.98
2.98	7.02
2.98	7.02
2.97	7.03
2.91	7.09
2.84	7.16
2.75	7.25
2.64	7.36
2.59	7.41
2.44	7.56
2.25	7.75

5.67	4.33
0	10
3.76	6.24
2.83	7.17
7.41	2.59
8.1	1.9
5.99	4.01
5.62	4.38
8.01	1.99
3.93	6.07
0	10
4.42	5.58
7.59	2.41
6.69	3.31

2.24	7.76
2.16	7.84
2.12	7.88
2.07	7.93
2.03	7.97
1.93	8.07
1.55	8.45
1.46	8.54
1.42	8.58
1.36	8.64
1.25	8.75
1.22	8.78
1.21	8.79
1.12	8.88
1.02	8.98
0.97	9.03
0.85	9.15
0.53	9.47
0.52	9.48
0.48	9.52
0.12	9.88
0.07	9.93
